# Fabrication of microcapsules with core‐shell structure for oral delivery of dual drugs and real‐time computed tomography imaging

**DOI:** 10.1049/nbt2.12058

**Published:** 2021-05-15

**Authors:** Rong Cai, Long Xiao, Miaomiao Zhang, Lulu Zhao, Jingjing Zhang, Fengyi Du, Zhirong Wang

**Affiliations:** ^1^ Zhangjiagang Hospital of Traditional Chinese Medicine Affiliated to Nanjing University of Chinese Medicine Zhangjiagang Jiangsu China; ^2^ School of Medicine Jiangsu University Zhenjiang China

## Abstract

Although multidrug combinations are an effective therapeutic strategy for serious disease in clinical practice, their therapeutic effect may be reduced because they conflict with each other medicinally in certain cases. Hence, there is an urgent need to develop a single drug carrier for precise multidrug delivery to avoid this interference. A reverse coordination method is reported that fabricates a double‐layer barium sulphate microcapsule (DL@BS MS) for two drugs separately loading simultaneously. In addition, BS nanoclusters were synthesised in situ inside the DL@BS MSs for real‐time computed tomography (CT) imaging. The results showed that the DL@BS MSs with a particle size of approximately 2 mm exhibited a uniform sphere. Because BS nanoclusters have a high X‐ray attenuation coefficient, the retention of DL@BS MSs in the digestive tract could be monitored through CT imaging in real time. More important, the core‐shell structure of DL@BS MSs encapsulating two different drugs could be released in spatiotemporal order in an acidic stomach environment. The as‐synthesis DL@BS MSs with a core‐shell structure and real‐time imaging performance provide an ideal carrier for the oral administration of multiple drugs simultaneously loaded but sequentially released.

## 1 INTRODUCTION

Oral administration means that drugs are absorbed into the blood by the digestive tract after they are taken orally and reach local or systemic tissues through blood circulation to achieve the purpose of treating disease [1]. It has the advantages of low cost [2], easy administration [3] and no direct damage to the skin or mucous membrane [4]. Nevertheless, most drugs are quickly discharged into the duodenum shortly after entering the stomach because of various factors such as gastric peristalsis and gastric emptying, which directly lead to a significant reduction in the bioavailability of drugs for stomach disease [5]. More important, some diseases, such as *Helicobacter pylori* infection, require two or more drugs for each treatment to achieve a curative effect. However, these drugs interfere with each other, seriously reducing the therapeutic effect. Therefore, it would make a lot of sense to avoid drugs interference and reduce the hassle of repeated administration in a short time.

Microencapsulation technology is a micropackaging technology for storing solids, liquids and gases [6, 7, 8], including physical, chemical, and physicochemical methods. The as‐prepared microcapsules (MSs) are miniature containers or packages with polymer or inorganic walls for oral drug delivery [9, 10, 11]. Growing evidence demonstrates that MSs are extensively applied in biomedical fields owing to their negligible toxicity, the isolation of active ingredients, and controlled release of contents [12, 13, 14]. Encouraged by these advantages, numerous researchers reported that oral administration of drugs loaded in MSs could protect the drugs and thus improve bioavailability. In our previous studies, alginate MSs with barium sulphate (BS) nanoclusters were constructed for probiotics delivery and real‐time computed tomography (CT) imaging [15]. Li's group fabricated dopamine MSs containing insulin to treat diabetes [16]. Hansen's group prepared MSs loading proclotting biotherapeutic factor VIII to transport and deliver haemostatic drugs [17]. Combination therapy involving two or more different drugs in one treatment is often required in the course of clinical drug treatment. When these drugs interference and conflict with each other, the traditional single structure of the carrier cannot meet the demand. Although each drug can be taken individually and continuously, this administration reduces the therapeutic effect and increases the operational complexity. Therefore, the development of a multidrug carrier for separate release temporally and spatially remains challenging.

**SCHEME 1:**
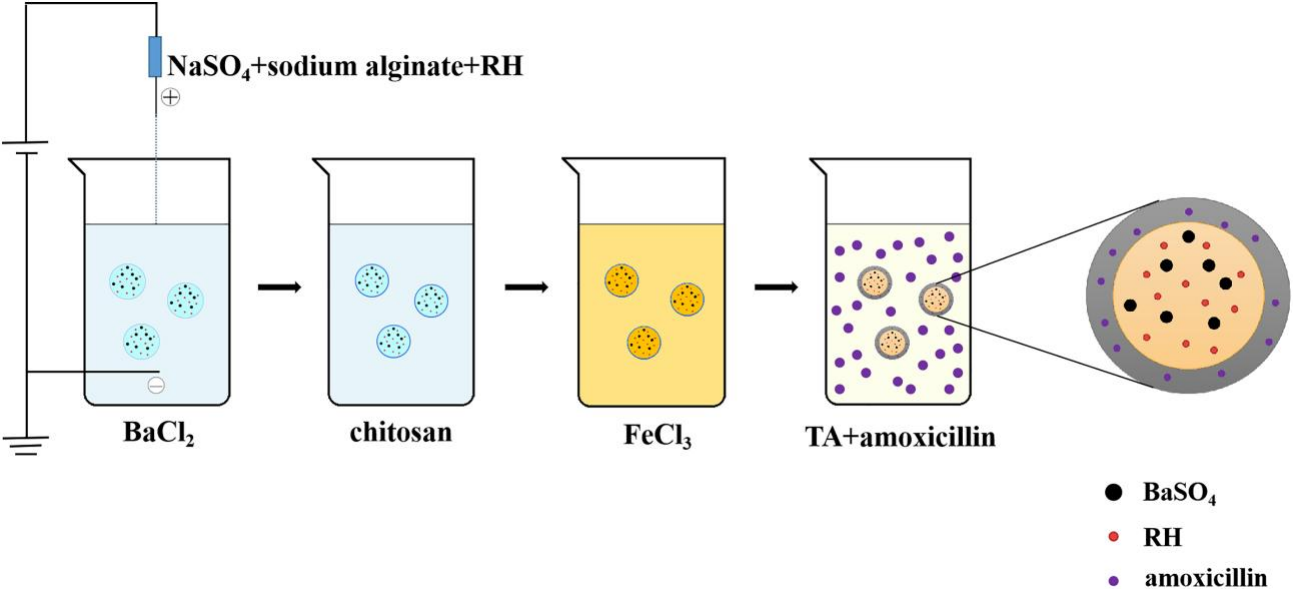
Preparation process of double‐layer barium sulphate microcapsules

To our best knowledge, few studies have mentioned loading multiple drugs into different substructures in one carrier for precise controlled release. In this study, we reported a reverse coordination method to fabricate core‐shell MSs with BS nanoclusters by a two‐step approach (Scheme 1). First, alginate microspheres with BaSO_4_ and ranitidine hydrochloride (RH) were prepared using an electrostatic spray method via the barium ion cross‐link alginate and sulphate ions simultaneously. Next, a shell with amoxicillin was formed on the surface of alginate microspheres through coordination interaction between iron ion and tannic acid (TA). The as‐synthesised DL@BS MSs contain two layers of relatively independent space, which make it possible to load and release drugs separately. RH and amoxicillin can both be used to treat gastric ulcers, so combined encapsulation of the two provides an idea for clinical application. In addition, BS nanoclusters formed in situ in the DL@BS MSs could be used as a CT contrast agent to detect the retention of MSs in real‐time in the digestive tract and evaluate drug delivery performance.

**FIGURE 7:**
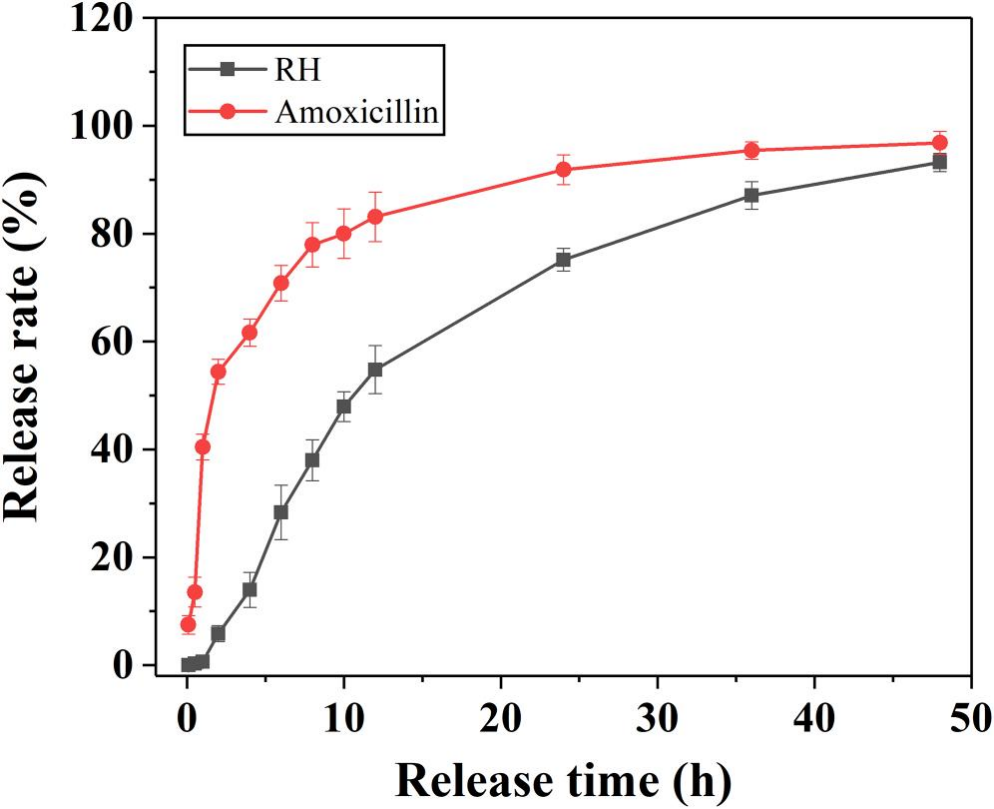
Cumulative release curves of ranitidine hydrochloride and amoxicillin from double‐layer barium sulphate microcapsules

## 2 MATERIALS AND METHODS

### 2.1 Materials

Barium chloride dihydrate and sodium sulfate anhydrous were purchased from Sinopharm Chemical Reagent Co., Ltd. Sodium alginate, chitosan, TA, ferric chloride hexahydrate, RH, amoxicillin, fluorescein diacetate and rhodamine B were obtained from Aladdin Industrial Inc. Microinjection pump was purchased from Zhejiang Smith Medical Instrument Co. Ltd. The phase structure was analysed by X‐ray diffraction (XRD) on a Rigaku‐D/MAX2500 diffractometer (Rigaku), equipped with a Cu Kα radiation source (λ = 0.15418 nm), with a slit of 0.5° and a scanning speed of 7° min^−1^, and X‐ray photoelectron spectroscopy (XPS) on Escalab 250Xi (Thermo Scientific). High‐performance liquid chromatography (HPLC) instrument was purchased from Shimadzu.

### 2.2 Synthesis of DL@BS MSs

DL@BS MSs were prepared by electrostatic spraying and reverse coordination in two steps. First, the alginate MSs loaded with BaSO_4_ and drug 1 were synthesised by electrostatic spraying at 20 V and a flow rate of 50 ml/h. During the process, a mixture of 1% sodium alginate, 0.5% of Na_2_SO_4_ and a certain amount of RH served as the injection solution and 4% BaCl_2_ solution served as the receiving solution. Second, the resultant white microspheres were immersed in a dilute acetic acid solution containing 0.2% chitosan, which was then wrapped with chitosan to make the structure more rigid. After 30 min, the microspheres were collected and immersed in 1% FeCl_3_ for 1 h. Owing to the high concentration of the external solution, Fe^3+^ could enter the MSs through free diffusion, forming single‐layer microspheres with full Fe^3+^ ion. Third, these microspheres were taken out and immersed into 0.5% TA and drug 2 solution. At this point, the high concentration of iron was going to spread out and coordinate with TA on surface of the microspheres, resulting in the formation of an Fe‐TA shell and loading amoxicillin. After 4 h, DL@BS MSs were obtained and stored in normal saline at 4°C for later use.

### 2.3 Characterisation of DL@BS MSs

The morphology and diameter of DL@BS MSs were observed using a digital camera and microscope in which the field of view was 10× and 40×, respectively. A scanning electron microscopy (Zeiss Merlin Compact) was also used to observe and compare the morphologic differences between single‐layer (SL)‐BS MSs and DL@BS MSs. The phase structure of DL@BS MSs was analysed by XRD on a Rigaku‐D/MAX2500 diffractometer(Rigaku), equipped with a Cu Kα radiation source (λ = 0.15418 nm) ranging from 5° to 75° with a 0.5° slit and a scanning speed of 7° min^−1^.

### 2.4 Biocompatibility of DL@BS MSs

A Cell Counting Kit‐8 (CCK‐8) assay (Solarbio) was used to evaluate the biocompatibility of DL@BS MSs by incubating with NIH 3T3 cells (a mouse embryonic fibroblast cell line) and RAW cells (mouse peritoneal macrophage cell line), respectively. We inoculated the two kinds of cells into 96‐well plates separately at 10^4^/well. When the cell density reached 80%, different amounts of DL@BS MSs (0, 5, 10, 20, 40, 80 and 160) were immersed into the medium. After overnight coincubation, cell viability was measured by CCK‐8 method.

### 2.5 Retention of DL@BS MSs in vivo

All animal experiments were killed according to the protocol approved by the Animal Management Rules of the Ministry of Health of the People's Republic of China and approved by the Institutional Animal Care and Use Committee of Jiangsu University. Kunming mice (male, age 6–8 weeks) were purchased from the Model Animal Genetics Research Centre of Jiangsu University. Mice were kept from food for 1 day before they were gavaged with 500 mg of DL@BS MSs. Then, the mice were scanned at a certain interval including 5 min and 1, 2, 4, 12, 24, 36 and 48 h by a 16‐slice spiral CT instrument (SOMATOM Sensation, Siemens) and the corresponding CT images were collected.

### 2.6 Biodegradation of DL@BS MSs detected by MR images in vitro

To investigate the magnetic resonance imaging (MRI) results, 10 mg of DL@BS MSs was immersed into 90 ml of simulated gastric juice. Then, the mixture was placed in a shaker at 37°C and 80 rpm/min rotating speed. DL@BS MSs and simulated gastric juices were collected after 0.5, 1, 2, 3, 4, 5, 6, 7  and 8 h, and were placed in a 96‐well culture plate in chronological order, and then a T1‐ and T2‐weighted scan was performed using a 3‐T MRI scanner (Magnetom Trio TIM Siemens).

### 2.7 Drug release test in vitro

The controlled release of RH and amoxicillin from DL@BS MSs was measured by HPLC. First, standard concentration–peak area curves were drawn by HPLC in which RH and amoxicillin were prepared at different concentrations of 0, 12.5, 25, 50, 100, 200 and 400 mg/ml, where the flow rate was 1 ml/min and the mobile phase was chromatography‐grade methanol. In addition, the DL@BS MSs loaded with two drugs were prepared by mixing 0.5% RH into the injection solution and 0.2% amoxicillin into the receiving solution during the preparation, in which RH was loaded in the inner layer and amoxicillin was loaded in the outer shell. Then, 10 mg of DL@BS MSs loaded with drugs was immersed into 90 ml of simulated gastric juice and the mixture was placed in a shaker at 80 rpm/min and 37°C. The simulated gastric juice was collected at 12 time points: 5 min and 0.5, 1, 2, 4, 6, 8, 10, 12, 24, 36  and 24 h, respectively. After filtered through 0.22‐μm filter membrane, the collected solution was measured by HPLC. Finally, the amount of corresponding drug release was calculated according to the standard curves of the two drugs. The drug release accumulation rate of RH or amoxicillin was calculated from the equation:

### 2.8 Statistical analysis

All data are presented as means ± SD. Statistical comparisons were statistically analysed by one‐way analysis of variance. *P* < 0.05 was considered statistically significant.

## 3 RESULTS AND DISCUSSION

### 3.1 Morphology of DL@BS MSs

**FIGURE 1:**
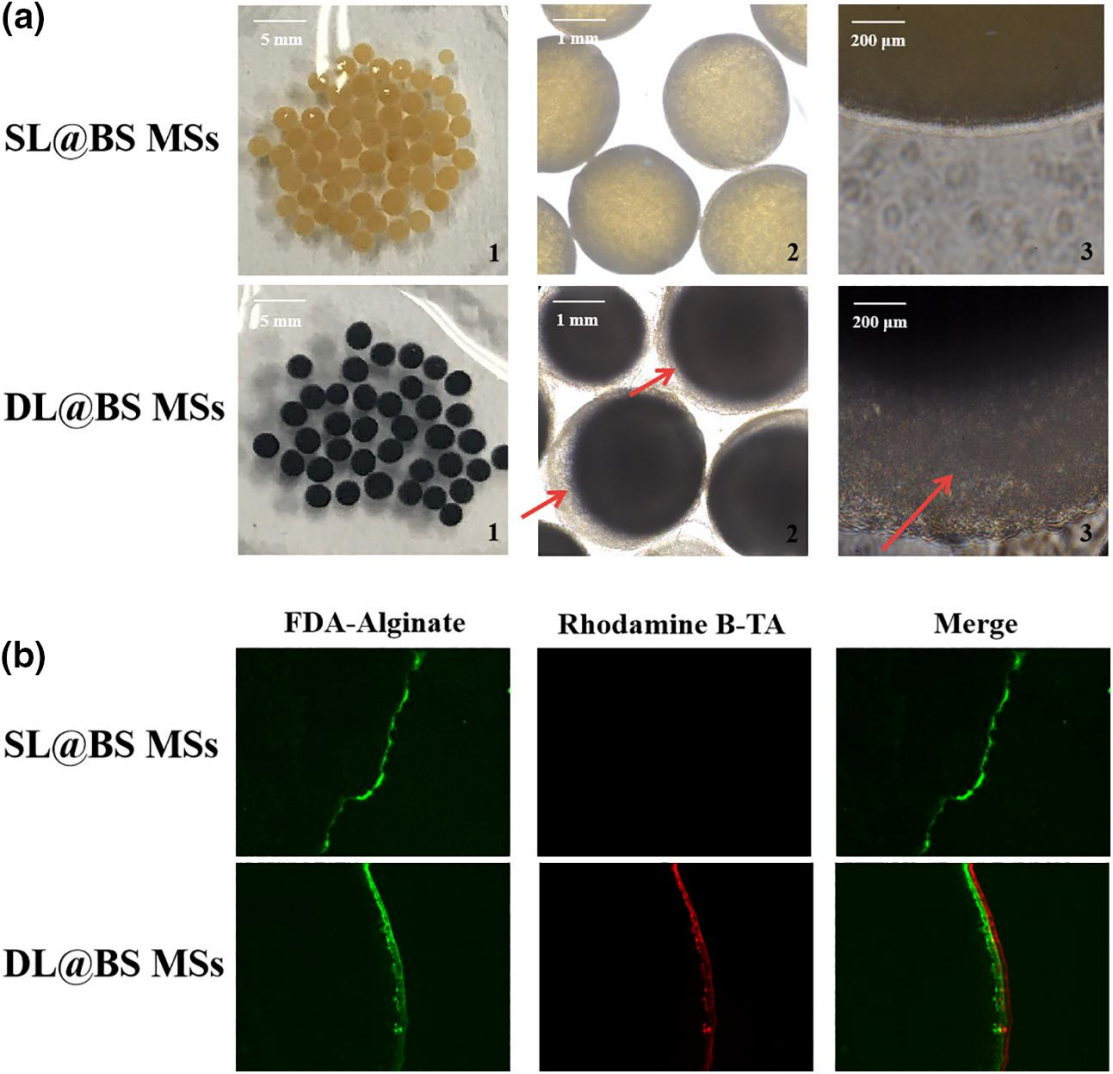
(a) Digital images of single‐layer barium sulphate microcapsules (SL@BS MSs) and double‐layer barium sulphate microcapsules (DL@BS MSs). (b) Fluorescent images of SL@BS MSs and DL@BS MSs

The DL@BS MSs with a core‐shell structure were successfully synthesised by electrostatic spray method and reverse coordination reaction. The morphology of DL@BS MSs was observed using a digital camera and fluorescence microscope. As shown in Figure 1(a), SL@BS MSs without a TA‐Fe^3+^ shell were pale yellow and exhibited a single structure of a solid ball. When coated with a TA‐Fe^3+^ shell, DL@BS MSs became black and displayed an obvious DL structure under white light. The obvious outer shell structure is shown with the red arrow. Furthermore, fluorescence images proved the presence of a thick shell and nucleus structure for DL@BS MSs. The alginate and TA were labelled with fluorescein diacetate and rhodamine B, respectively. Next, the prepared SL@BS MSs and DL@BS MSs from fluorescent=labelled molecules were cut into slices and were observed by fluorescence microscopy. As shown in Figure 1(b), only green fluorescence was observed in SL@BS MSs, whereas two kinds of green and red fluorescence were observed in DL@BS MSs. In addition, the red fluorescence almost adhered to the outer edge of the green fluorescence. Alginate was the inner material of microspheres, whereas TA was the outer material. The fluorescent images indicated the existence of the core‐shell structure of DL@BS MSs, which was consistent with our expectation. From these data, we conclude that DL@BS MSs with an inner layer of sodium alginate and an outer shell of a TA‐Fe^3+^ cross‐linked product were successfully prepared.

### 3.2 Characterisation of BaSO_4_ in DL@BS MSs

**FIGURE 2:**
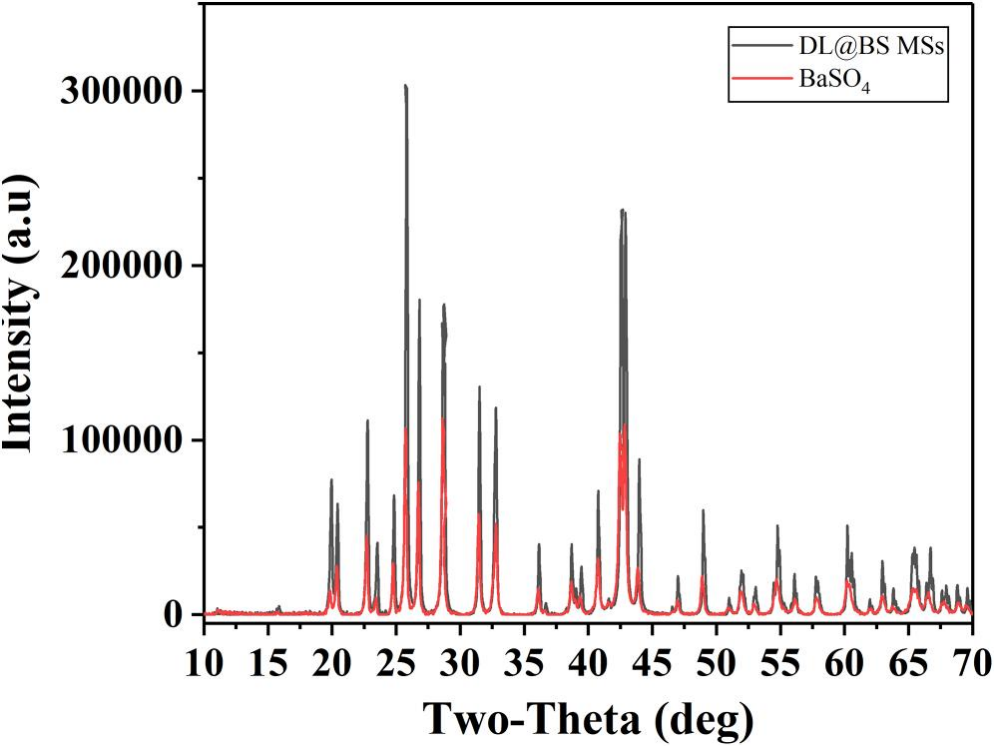
X‐ray diffraction pattern of double‐layer barium sulphate microcapsules and BaSO_4_precipitate

During the electrostatic spray, the receiving solution contained BaCl_2_ and the injected solution contained NaSO_4_. Therefore, BaSO_4_ nanoclusters were synthesised in situ in DL@BS MSs when the injected solution sprayed into the receiving solution. The phase structure of as‐prepared BaSO_4_ nanoclusters inside DL@BS MSs was investigated by XRD. As shown in Figure 2, XRD spectra showed that there were three sharp diffraction peaks at 26°, 28° and 43°, demonstrating that BaSO_4_ nanoclusters inside DL@BS MSs had a good crystal structure. In addition, BaSO_4_ precipitate with good biocompatibility and a high X‐ray attenuation coefficient were successfully applied in CT imaging as a contrast agent for the diagnosis of gastrointestinal disease. Hence, BaSO_4_ nanoclusters could provide for real‐time CT imaging to detect the retention of DL@BS MSs in the digestive tract.

### 3.3 Chemical properties of DL@BS MSs

**FIGURE 3:**
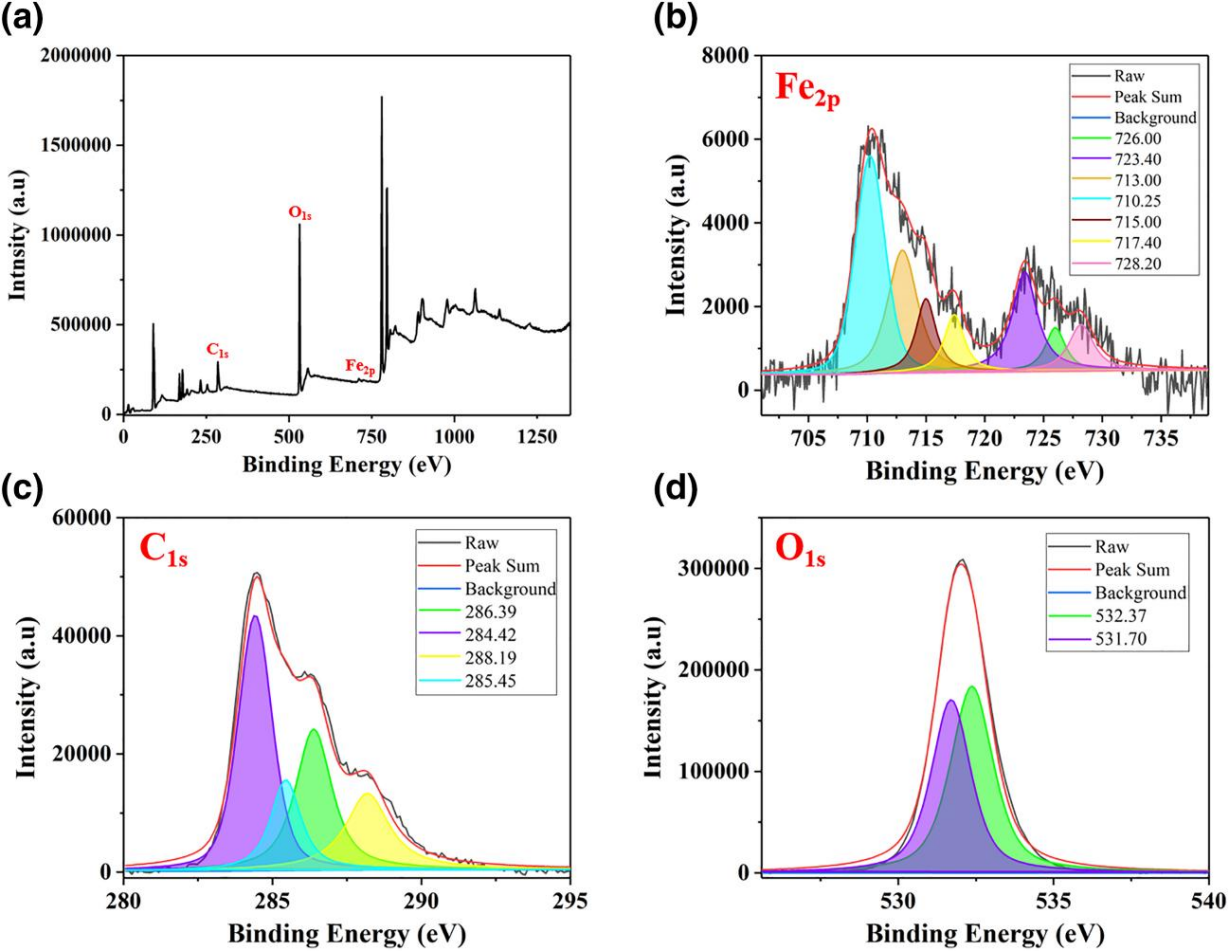
Full‐scan X‐ray photoelectron spectroscopy spectrum of (a) double‐layer barium sulphate microcapsules; (b) Fe_2p_ spectrum, (c) C_1s_ spectrum and (d) O_1s_ spectrum

The surface chemical structure of DL@BS MSs was analysed by XPS. As shown in Figure 3(a), there were three peaks at 285, 532, and 711 eV respectively, which corresponded to three elements (carbon, oxygen and iron), proving the elemental composition of DL@BS MSs. As shown in Figure 3(b), the presence of peaks at 710.25, 713.00, 715.00, 717.40, 723.40, 726.00 and 728.20 eV demonstrates the existence of Fe^3+^ iron in DL@BS MSs. The C_1s_ spectrum (Figure 3(c)) revealed four carbon species, 284.42, 285.45, 286.39 and 288.19 eV, which indicated the existence of carbon atoms. Moreover, as shown in Figure 3(d), the spectrum of O_1s_ showed two obvious peaks at 531.70 and 532.37 eV. These data are consistent with our expectations, which proves that we successfully prepared DL@BS MSs containing Fe^3+^.

### 3.4 CCK‐8 assay

**FIGURE 4:**
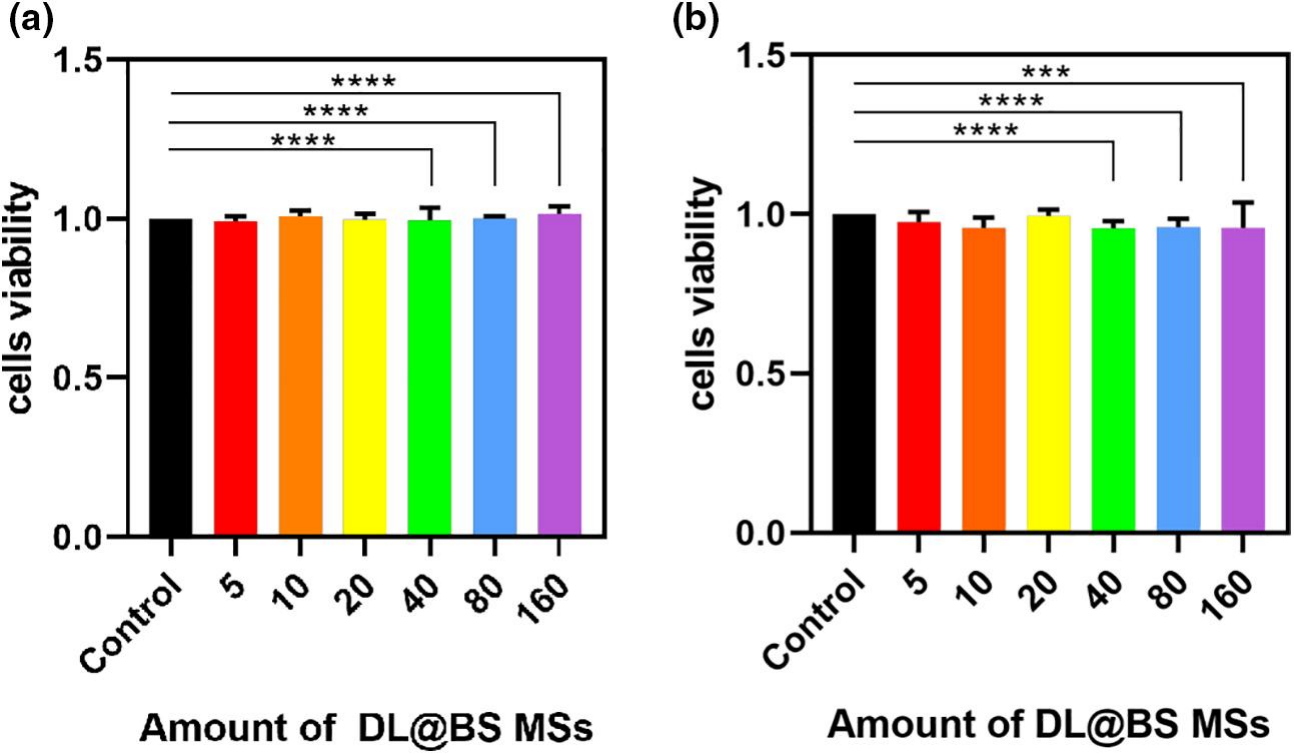
Effect of double‐layer barium sulphate microcapsules on cell viability of (a) NIH 3T3 cells and (b) RAW cells

Owing to their capability of drug‐loading, DL@BS MSs are expected to be applied as drug carriers for oral administration; thus, their biocompatibility is the first thing we should consider and explore. We assessed the cytotoxicity of DL@BS MSs using a CCK‐8 assay. The cell viability of NIH 3T3 cells and RAW cells was measured after 24 h exposure to different amounts of DL@BS MSs (0, 5, 10, 20, 40, 80 and 160 per hole). As shown in Figure 4, the cell viability of the two kinds of cells at various amount of DL@BS MSs was nearly 100%, which indicated that the prepared DL@BS MSs had good biocompatibility.

### 3.5 Real‐time tracking of DL@BS MSs by CT imaging in vivo

**FIGURE 5:**
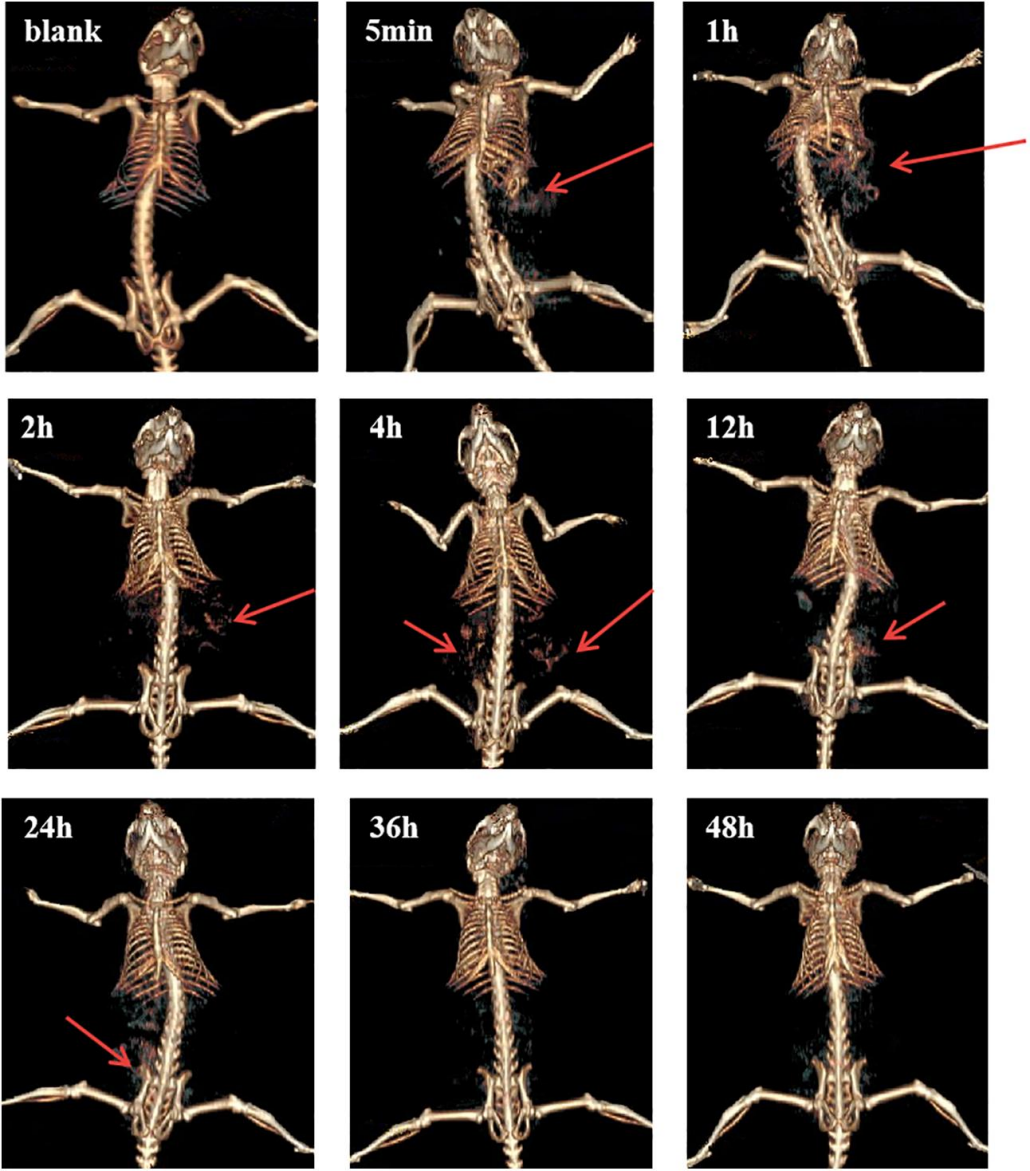
Computed tomography images of the digestive tract of mice after oral double‐layer barium sulphate microcapsule administration

The retention of DL@BS MSs in the digestive tract after oral administration was evaluated by CT imaging. After oral administration of DL@BS MSs, mice were scanned by CT scanner at different time points from 5 min to 48 h, and three‐dimensional renderings of CT images were collected. As shown in Figure 5, the CT signal of the stomach could be observed 5 min after administration, which contributed to the existence of DL@BS MSs in the stomach. After 1 h of administration, a small amount of CT signal could be detected in the small intestine owing to the displacement of DL@BS MSs in the digestive tract, whereas the CT signal in the stomach remained strong. This process continued until 2 h after administration, and DL@BS MSs were completely discharged from the stomach into the small intestine. The CT signal in the small intestine was weakened 4 h after administration, whereas the CT signal in the large intestine was enhanced, indicating that DL@BS MSs had gradually emptied from the small intestine to the large intestine. DL@BS MSs were almost discharged into the large intestine 12 h after administration and very little remained in the small intestine. After 24 h of administration, CT signals could hardly be detected in the small intestine, and there were fewer DL@BS MSs in the digestive tract than when gavage had just begun. This result indicated that DL@BS MSs had gradually emptied from the body of the mice. No CT signal was detected at either time point of 36 or 48 h, which meant that DL@BS MSs had been completely expelled from the mice. The distribution and quantity of DL@BS MSs in the digestive tract of mice are shown by the red arrow. These data show that DL@BS MSs had good retention and imaging capabilities in the digestive tract in mice, which provide the possibility for DL@BS MSs carrying drugs for oral administration to treat disease.

### 3.6 Magnetic resonance imaging and biodegradation of DL@BS MSs in vitro

**FIGURE 6:**
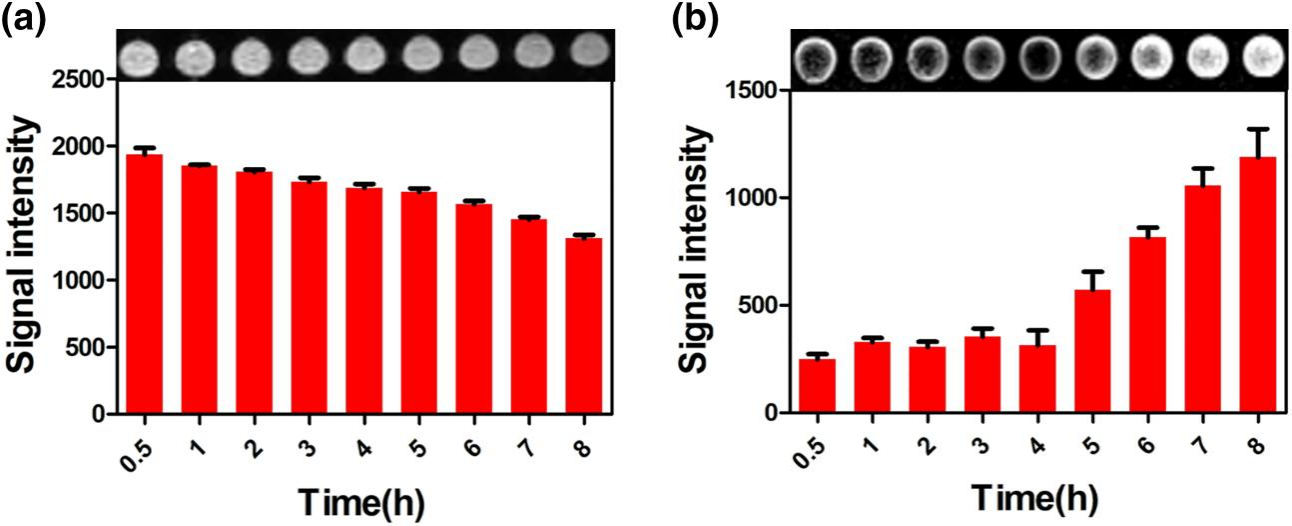
Magnetic resonance images of (a) double‐layer barium sulphate microcapsule formation and (b) degradation under simulated gastric juice

Owing to the presence of phenolic hydroxyl groups, TA can complex with iron ions to form a blue‐black polymer. According to this principle, we prepared the Fe‐TA shell of DL@BS MSs by osmosis. Moreover, iron polymer is a good contrast agent for MRI; thus, we speculated that the biodegradation and stability of DL@BS MSs could be detected by an MR signal from the Fe‐TA chelating complex. DL@BS MSs were immersed into simulated gastric juice and collected at nine different time periods including 0.5, 1, 2, 3, 4, 5, 6, 7 and 8 h, respectively. The Fe‐TA chelating complex tended to degrade under acidic condition, leading to a reduction in the MR signal. As shown in Figure 6(a) with the prolongation of cross‐linking time, the intensity of MR signal in DL@BS MSs decreased gradually according to iron‐mediated T2‐weighed imaging, indicating the formation of an Fe‐TA chelating complex. When the soaking solution was collected at each time point, the signal intensity increased with incubation time under acidic conditions, suggesting the degradation of the Fe‐TA chelating complex (Figure 6(b)). The Fe‐TA chelating complex remained relatively stable within 4 h and then accelerated in degradation. This feature was conductive to protecting the carrier drug when passing through the stomach and arriving at the intestines.

### 3.7 Drug release profiles of DL@BS MSs in vitro

To test the drug release performance, two drugs (RH and amoxicillin) were loaded into the core and shell of DL@BS MSs, respectively. Specifically, RH was mixed with the injected solution and loaded in the core during electrostatic spraying, while amoxicillin was mixed with accepted solution and loaded in the shell of cross‐linked products of TA and Fe^3+^. The drug‐loaded DL@BS MSs were immersed into the simulated gastric juice, and then release of the two drugs from DL@BS MSs was measured in real time by HPLC. As shown in Figure 7, the cumulative drug release of RH was 91.85% and 75.17%, whereas amoxicillin was 96.36% and 93.22% at 48 and 24 h, respectively. Generally, both drugs showed a similar tendency of releasing quickly and then slowly in simulated gastric juice, but the cumulative release of amoxicillin was higher than that of RH at the same time point. This phenomenon corresponded to the core‐shell structure of DL@BS MSs, where amoxicillin was loaded in the shell and RH was loaded in the core. These results demonstrated that DL@BS MSs made it possible to load and release the two different drugs hierarchically, which provided ideal technical support for the clinical concurrent use of two drugs with opposite drug properties.

## 4 CONCLUSIONS

We successfully prepared DL@BS MSs with a core‐shell structure by electrostatic spray and reverse coordination technology. The core of DL@BS MSs was formed via crosslinked alginate whereas the shell was formed bu a coordination reaction between Fe^3+^ and TA. The results demonstrated that this core‐shell structure was conductive to the loading and release of two drugs step by step, which avoided conflicts with each other. In addition, BaSO_4_ nanoclusters were synthesised in situ in the core of the DL@BS MSs and served as an ideal CT contrast agent for real‐time imaging. Overall, this DL@BS MSs could be used as a multifunctional carrier for oral multidrug delivery and real‐time imaging.
